# Cognitive Functions following Trigeminal Neuromodulation

**DOI:** 10.3390/biomedicines11092392

**Published:** 2023-08-27

**Authors:** Beniamina Mercante, Paolo Enrico, Franca Deriu

**Affiliations:** 1 Department of Biomedical Sciences, University of Sassari, 07100 Sassari, Italy; bmercante@uniss.it (B.M.); enrico@uniss.it (P.E.); 2 AOU Sassari, Unit of Endocrinology, Nutritional and Metabolic Disorders, 07100 Sassari, Italy

**Keywords:** trigeminal nerve, neuromodulation, cognition, peripheral nerve stimulation, transcutaneous trigeminal nerve stimulation

## Abstract

Vast scientific effort in recent years have been focused on the search for effective and safe treatments for cognitive decline. In this regard, non-invasive neuromodulation has gained increasing attention for its reported effectiveness in promoting the recovery of multiple cognitive domains after central nervous system damage. In this short review, we discuss the available evidence supporting a possible cognitive effect of trigeminal nerve stimulation (TNS). In particular, we ask that, while TNS has been widely and successfully used in the treatment of various neuropsychiatric conditions, as far as research in the cognitive field is concerned, where does TNS stand? The trigeminal nerve is the largest cranial nerve, conveying the sensory information from the face to the trigeminal sensory nuclei, and from there to the thalamus and up to the somatosensory cortex. On these bases, a bottom-up mechanism has been proposed, positing that TNS-induced modulation of the brainstem noradrenergic system may affect the function of the brain networks involved in cognition. Nevertheless, despite the promising theories, to date, the use of TNS for cognitive empowering and/or cognitive decline treatment has several challenges ahead of it, mainly due to little uniformity of the stimulation protocols. However, as the field continues to grow, standardization of practice will allow for data comparisons across studies, leading to optimized protocols targeting specific brain circuitries, which may, in turn, influence cognition in a designed manner.

## 1. Introduction

During the last two decades, the evolution and expansion of neuromodulation techniques have made them among the most attractive non-pharmacological tools for selected neurological and neuropsychiatric disorders. In fact, with respect to conventional drug-based approaches, neuromodulation techniques often provide several advantages such as safety, flexibility, and (in some instances) specificity of the treatment [1,2,3,4,5,6,7].

Nowadays, the panorama of non-invasive neurostimulation includes several techniques, which are thought to operate by stimulating the brain directly (e.g., transcranial direct current stimulation, tDCS; repetitive transcranial magnetic stimulation, rTMS; transcranial ultrasound stimulation, TUS, etc.). Interestingly, recently developed TUS allows for a focused application of the stimulus, overcoming a serious limitation of the other non-invasive techniques, namely their low spatial resolution [5,8]. On the other hand, stimulation of peripheral nerves (e.g., vagal nerve stimulation, trigeminal nerve stimulation, etc.) is also an effective non-invasive neurostimulation technique. With respect to direct neurostimulation techniques, peripheral nerve stimulation is often more practical and cheaper, while maintaining a relevant therapeutic power.

In recent times, there has been growing interest in the use of neuromodulation approaches to modulate the activity of the healthy brain, with the general aim of overcoming the physiological limitations of human capabilities, both physical and cognitive [9]. Applications may range from the enhancement of military personnel’s mental and/or physical skills [10,11], to the treatment of cognitive decline, either aging- or neurodegeneration-related, which significantly impacts the daily life of millions of people worldwide, urging the necessity for novel treatments [12,13]. While the majority of the studies in the field have been performed using tDCS, in recent years, it has been shown that cranial nerve stimulation may also affect brain circuits linked to cognitive domains [3,14,15]. Among all peripheral neuromodulation techniques, transcutaneous trigeminal nerve stimulation (TNS) has recently gained increasing success in clinical practice, since it has been consistently proven to exert beneficial effects in the symptomatic treatment of several neuropsychiatric disorders [16,17].

However, the real therapeutic potential, as well as the mechanism(s) of non-invasive neuromodulation methods on cognitive performance in both pathological conditions and healthy individuals, remains unclear. In this short review, we will focus on the current knowledge of the neurobiological mechanisms by which TNS may affect cognition.

## 2. Trigeminal Nerve Stimulation: Use and Safety

The first evidence of efficacy of TNS in an animal model of pentylenetetrazole-induced convulsions dates back more than twenty years ago [18]. Indeed, TNS was originally developed mainly as an alternative to vagal nerve stimulation (VNS) to overcome its well-known limitations. In particular, these include VNS-induced autonomic side effects due to the presence in the vagus nerve of visceral efferent components affecting cardiac and laryngeal function [19,20], and the exposure to surgery-related risks such as peritracheal hematoma and infections [21,22]. Since then, TNS has been tested in the treatment of different neurological and psychiatric conditions, proving beneficial for seizure control, depression, migraine, attention deficit hyperactivity disorder (ADHD), post-traumatic stress disorder, and tinnitus [23,24,25,26,27,28,29,30,31,32,33,34,35,36,37,38,39,40,41]. TNS has now reached a considerable diffusion in clinical practice, allowing for the development of a substantial base of knowledge supporting its possible therapeutic applications. Reported side effects are generally mild and include drowsiness, skin irritation, and headaches [4,7] However, increased appetite, fatigue, trouble sleeping, and teeth clenching have also been reported in ADHD patients [29]. In short, the available evidence shows that TNS administration is well tolerated, and no severe adverse events occur after treatment [27,42]. In April 2019, the U.S. Food and Drug Administration (FDA) allowed the marketing of the first medical TNS device to treat ADHD [36], and, as of today, TNS remains the first non-drug treatment for ADHD granted marketing authorization. The FDA has also approved the use of TNS for the treatment of migraine [43,44], and several clinical trials for drug-resistant epilepsy, depression, and other conditions are in different stages of completion. To date, in Europe, TNS has obtained approval for adjunctive treatment of epilepsy and major depressive disorder for patients over nine years of age [45,46]. Interestingly, besides being approved for epilepsy, depression, and ADHD therapy, TNS has been shown to have the potential to enhance cognition and mood in both healthy subjects and patients with neuropsychiatric disorders [41,47,48].

**Figure 1:**
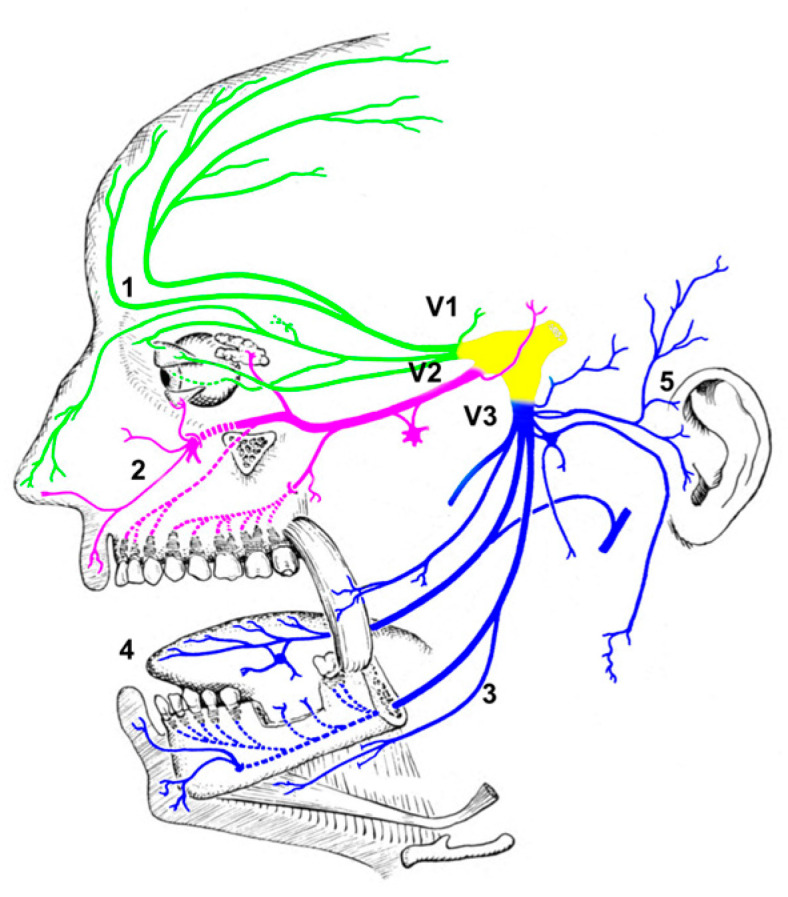
Trigeminal nerve branches shown in three different colors: V1—Ophthalmic branch (green); V2—Maxillary branch (pink); and V3—Mandibular branch (blue). Numbers show the electrode placement over the peripheral branches of the trigeminal nerve used in key studies on trigeminal nerve stimulation and cognition: 1—Supraorbital nerve; 2—Infraorbital nerve; 3—Motor branch; 4—Lingual nerve; and 5—Auricolo-temporal nerve.

Along with the favorable safety profile of TNS, its ease of use is certainly an important factor favoring its wide diffusion. In fact, nowadays, technological advancements allow the production of portable, affordable, and user-friendly external stimulators. A TNS device is generally constituted by a programmable pulse generator delivering low-intensity electrical pulses to small electrodes applied to the cutaneous areas overlying specific branches of the trigeminal nerve. This allows great ease and flexibility in the administration of TNS, ranging from the application of disposable patches for overnight stimulation to the use of simple wearable devices. Furthermore, the trigeminal nerve may also be stimulated via the auricular branch, with the advantage of stimulating electrodes being less noticeable. This may allow for longer stimulation protocols, which can also be continuous throughout the day, including working hours. TNS devices are also affordable, and their use does not require trained operators, contrary to the use of other neurostimulation techniques (such as rTMS or tDCS), which often involve a more complex instrumental setting, as well as specialized personnel. Typically, TNS stimulation electrodes are positioned on the forehead branches of the first (V1) or second (V2) division of the trigeminal nerve (i.e., the supraorbital and infraorbital nerves, respectively). Other positioning schemes are possible (such as the anterosuperior and medial areas of the external ear innervated by the auricolo-temporal nerve, or the masseter belly innervated by the trigeminal motor branches), and may even result in other benefits, although we know little about them (Figure 1). In fact, mainly due to the lack of knowledge of the neurobiological bases of the effects of TNS, right now, the choice of stimulation parameters (waveform, amplitude, frequency, pulse duration, duty cycle, and session duration), and electrode placement heavily depends on the device characteristics and the physician’s particular experience for the condition to be treated.

## 3. How Trigeminal Nerve Stimulation May Affect Cognitive Function

Cognition has been classically defined as “all the processes by which the sensory input is transformed, reduced, elaborated, stored, recovered and used” [49]. While more recent efforts are still trying to reach a better definition of cognition [50], it is now largely accepted that cognitive processes emerge from the coordinated activity of hundreds of brain regions [51,52,53]. Brain network activity is dynamically reconfigured according to contingent sensory inputs, and the resulting state of the network involved is thought to produce a specific result [54,55]. This allows the brain to perform a large variety of cognitive and motor tasks, in order to adapt to changing environments.

Cranial nerves are a specialized part of the peripheral nervous system conveying crucial sensory information from and to the brain. Their inputs can modulate regional brain activity, potentially affecting the respective cognitive functions [3], as well as the activity of the brain networks they are involved in. Depending on the stimulated structure and stimulation modality, the result can either be inhibitory or facilitatory, thus influencing brain network activity in different ways. All these effects have been attributed to a “bottom-up” mechanism of modulation of the signal processing performed by the central nervous system (CNS), which is also thought to be the basis of the effects of TNS [6,56,57]. The trigeminal nerve is the largest cranial nerve, conveying the sensory information from the face, a major part of the scalp, the teeth, the oral and nasal cavity, as well as providing the motor supply to the masticatory muscles, the mylohyoid, the tensor tympani and tensor *veli palatini* muscles [58]. Trigeminal sensory nuclei send sensory information to the thalamus and from here to the somatosensory cortex [59], but also project to multiple nearby brainstem nuclei [60] including the nucleus solitary tract (NST), the locus coeruleus (LC), and the raphe nucleus (RN). As a whole, the output of the trigeminal nuclei can influence the activity of several higher-level structures. The influence on the NST appears particularly relevant since this structure integrates visceral inputs and relays signals to various structures such as the LC, RN, the reticular activating system hypothalamus, thalamus, amygdala, hippocampus, limbic forebrain, anterior cingulate cortex, and insula [60]. These pathways also open the possibility of regulating attention, cognition, and sensorimotor behaviors using the TNS via a bottom-up mechanism [48]. However, so far, the effects of TNS on cognitive processes have been explored only within neurological and neuropsychiatric populations, and only a few studies specifically investigated TNS effects on cognition as a stand-alone phenomenon. 

In laboratory animals, the neuroanatomical basis and possible sites for action of TNS in physiological conditions have been poorly investigated so far. In the work by Mercante and colleagues [61], it has been shown that acute TNS administration in male adult Sprague Dawley rats increases c-Fos-like immunoreactivity in selected brainstem and forebrain regions. In further detail, following 3-hour-long unilateral stimulation of the infraorbital nerve (IoN, V2 division), a significant increase in c-Fos-like immunoreactivity was observed not only in the trigeminal sensory nuclei and in the somatosensory cortex, as expected, but also in the NST, RN, LC, amygdala, endopiriform nucleus, hippocampus, and entorhinal cortex. Such a diffuse effect, even after acute short-term TNS administration, supports the postulate that TNS can have a widespread influence on higher subcortical and cortical structures. The same study demonstrated that cell proliferation in the dentate gyrus of the hippocampus (as indexed by bromodeoxyuridine cell incorporation) was consistently increased after 24 h from acute TNS administration, in comparison to sham and naïve rats. Interestingly, since it has been proposed that the increase in hippocampal cellular proliferation may be due to an increase in noradrenergic activity [62,63,64], the marked c-Fos-like immunoreactivity observed in the LC after TNS may be causally related to it. Furthermore, in a subchronic pilocarpine-based epilepsy model (4 weeks of treatment, percutaneous stimulation of the IoN) TNS reduced hippocampal apoptosis and inflammation, showing protective effects on cognitive functions such as learning and memory [65].

The evidence provided in experimental animals gives support to the idea that the trigeminal system has the full potential to influence the activity of cortical and subcortical structures involved in cognitive, affective, and behavioral control [61,66]. Nevertheless, available human studies on TNS effects on cognition and behavior are still rather inconclusive (Table 1).

The effect(s) of TNS on CNS activity in healthy human subjects have been investigated using different non-invasive methods such as transcranial magnetic stimulation (TMS), electroencephalography (EEG), magnetic resonance spectroscopy, and brainstem reflex recordings. Paired-pulse TMS was used to evaluate the activity of inhibitory and excitatory intracortical circuits in the hand representation area of the primary motor cortex, during and after acute TNS administration in healthy subjects [67,68]. These studies failed to demonstrate significant changes in any of the neurophysiological outcomes tested (i.e., motor threshold, amplitude of the TMS-evoked motor potential, short-latency intracortical inhibition, and intracortical facilitation), suggesting that TNS effects may be exerted mainly at the brainstem level [67,68]. This hypothesis was also supported by other studies showing that short-term TNS administration significantly depressed brainstem circuits mediating the R2 component of the blink reflex [68], with a long-lasting action resembling long-term depression-like plasticity [69]. In agreement with this hypothesis, a recent paper has shown that short-term TNS was able to depress the amplitude of the defensive hand blink reflex [70], which is mediated by a brainstem circuit that is continuously modulated by cortical areas devoted to encoding the peripersonal space [71]. Ginatempo and colleagues [72] evaluated the effects of acute TNS administration at the cortical level using EEG, showing that TNS induces a spatially diffused, non-specific desynchronization of fast EEG rhythms, with a significant decrease in the intra- and inter-hemispheric synchronization of beta frequencies. In addition, a trend of increase in the gamma band power and in mean EEG frequency total power and a trend of decrease in interhemispheric gamma coherence was also observed during and after TNS. These results were interpreted as expressions at the cortical level of modifications originating in the brainstem, such as a change in the activity of the noradrenergic nuclei, namely LC [72]. Interestingly, a recent paper using ultrahigh-field magnetic resonance spectroscopy showed that acute TNS administration slightly decreased total creatine concentrations in the dorsolateral prefrontal cortex (DLPFC) ~60 min post-TNS [73]. However, while creatine CNS concentration has been shown to negatively correlate with measures of cognitive function [74], whether relatively small changes (4–5% decrease) in the DLPFC could provide a cognitive benefit is unknown. Vasomotor centers in the brainstem are directly connected to the trigeminal nerve [28,75,76], and its stimulation has a significant impact on cerebral perfusion [77,78,79]. Neurogenic control of cerebral blood flow via TNS may be a way to induce cerebral vasodilation and improve cerebral perfusion in both normal conditions and pathologic states [80,81]

On the behavioral side, studies investigating TNS effects in healthy subjects often reported a state of relaxation or sedation, decreased attention and vigilance, with a tendency to fall asleep during and/or immediately after acute TNS administration. Piquet et al. 2011 [82] investigated the sedative effects of TNS and reported a decrease in vigilance and arousal following acute high-frequency TNS. The authors hypothesized that a change in activity levels of the orexin-arcuate-periaqueductal gray matter circuit could occur during supraorbital neurostimulation and might explain the decrease in vigilance [82]. A subsequent study tested whether the sedative effects of TNS were associated with hypnotic effects, such as sleep latency reduction, and found no association with a reduction in sleep latency as indexed by the Multiple Sleep Latency Test [83].

Long-term treatment of TNS using low-frequency stimulation may also have a calming effect and improve sleep quality and mood [48]. A potential explanation for the ability of trigeminal modulation to influence sleep is the modulation of the ascending reticular activating system [84], a network of nuclei that control attention and the sleep/wake cycle including the LC and pedunculopontine nucleus [85]. 

Recently, the TNS effects on cortical activity related to modulation of the function of the noradrenergic system were also investigated using event-related potentials (ERP). This approach seems particularly relevant since many ERP components have been operationally related to several specific neurocognitive processes [86,87], including attention, working memory, and decision-making [88,89,90]. Both studies used short-term TNS administration but, interestingly, one study [91] used bilateral transcutaneous stimulation of the trigeminal motor branches (over the masseter belly, incisura sigmoidea), while in the other [92], the IoN sensitive branch was bilaterally stimulated.

In the first study, Fantozzi and colleagues [91] showed in 13 healthy subjects a reduction of the amplitude of the P300 wave elicited by an acoustic oddball paradigm in several cortical areas, and a positive correlation between P300 amplitude in frontal and median cortical region and pupil size. The authors ascribed these results to increased cortical norepinephrine (NE) levels due to a TNS-augmented activity of LC neurons. High cortical NE levels may improve sensory processing, suggesting that TNS could be used for improving cognitive performance in subjects with cognitive disorders or arousal dysfunction [91,93].

The second study focused on several major ERP components recorded during the administration of a simple visual oddball task (P200 and P300), and a paired-click paradigm (P50, N100, and P200), to investigate in healthy subjects the possible mechanism(s) of action of TNS related to high-level neurocognitive processes [92]. These paradigms are largely used to study information processing and cognitive brain functions [90,94], which are modulated by multiple brain systems and, in particular, by the LC and the reticular formation [95,96], both targeted by TNS [61]. Results show that all parameters measured were unaffected by TNS, as shown by the non-significant differences between the effects of sham and real TNS administration.

**Table 1:** Studies on the effects of trigeminal nerve stimulation report specific cognitive measures. Parameters and data as stated in the original publication.

*Citation*	*Study Population*	*Stimulated Nerve*	*TNS Parameters*	*Time of Assessment*	*Assessment Methodology*	*Outcome*	*Side Effects*
Piquet et al., 2011 [82]	HS	Supraorbital nerve, bilateral	(A) 120 Hz pulsed, 250 μs pulse width. (B) <14 mA (C) 20 min	After stimulation	Psychological test	Modulation of vigilance	No major side effects reported
Boasso et al., 2016 [48]	HS	Right supraorbital nerve, Cervical nerve	(A) biphasic pulse-modulated (3–11 kHz) (B) 5–7 mA (C) 20 min per day	After one week of treatment	Psychological test Biochemical measurements	Improved sleep quality, stress reduction	No major side effects reported
Trevizol et al., 2016 [40]	NPD	Supraorbital nerve, bilateral	(A) 120 Hz pulsed, 200 μs pulse width (B) N/A (C) 30 min per day	After 10 days of treatment	Psychological test	No significant difference in cognitive performances	No major side effects reported
Loo et al., 2020 [36]	ADHD	Supraorbital nerve, bilateral	(A) 120 Hz pulsed, 250 μs pulse width (B) 2–4 mA (C) 8 h per night	After 4 weeks of treatment	Psychological test Resting state EEG	Improved/normalized executive functions, modulation of right frontal brain activity	Increase in fatigue, headache, appetite. Skin whitening/discoloration under electrode patch
Tramonti Fantozzi et al., 2020 [93]	HS	Mandibular motor branch	(A) biphasic, 0.618 Hz, 540 μs pulse width, (B) 21–25 mA (C) 15 min	After stimulation	Psychological test Pupil size	Trigeminal sensory-motor imbalance may affect cognitive performance	No major side effects reported
Tramonti Fantozzi et al., 2021 [91]	HS	Mandibular motor branch	(A) biphasic, 0.618 Hz, 540 μs pulse width, (B) 21–25 mA (C) 10 min	After stimulation	Pupil size EEG power change ERP (auditory oddball)	Reduced P300 amplitude, positive correlation between P300 and pupil size	No major side effects reported
Mercante et al., 2023 [92]	HS	Infraorbital nerve, bilateral	(A) biphasic,120 Hz, 250 μs pulse width, (B) 1–20 mA (C) 20 min	After stimulation	ERP (visual oddball, sensory gating)	No changes in ERP parameters measured	No major effects reported

Healthy subjects: HS; Neuro-Psychiatric Disorders: NPD; Attention Deficit Hyperactivity Disorder: ADHD. TNS parameters: A: Stimulus characteristics; B: Stimulus intensity; C: Treatment duration. Electroencephalography: EEG; Event-Related Potentials: ERP.

## 4. New Possibilities

Ease of use and safety of TNS allows its administration in conjunction with other interventions. In particular, in recent years, the association of TNS with auricular or tongue stimulation has seen a growing interest and development [97,98,99].

As of today, auricular stimulation is mainly used for transcutaneous VNS (tVNS) via the auricular branch of the vagus nerve. However, the external ear is also innervated by trigeminal nerve fibers, partially overlapping with the vagus territory [100,101]; therefore, an appropriate electrode positioning easily results in the stimulation of both trigeminal and vagal afferents [6,102]. The study of auricular tVNS has seen great development in recent years, especially with regard to improved cognitive performance, not only in the clinical population but also in healthy subjects [103,104,105,106,107]. While the neurobiological mechanisms are still unclear, the available literature mostly seek to explain the positive effects of tVNS on cognitive performance in terms of a change in brain NE levels [64,108]. Thus, vagal afferents arriving at the NTS and then at the LC may cause an increase in NE levels, which would lead to an increase in brain activation and consequently to increased cognitive performance [109,110,111,112]. Consistent evidence shows that both trigeminal and vagal nerve systems share common relay stations in the brainstem, the spinal trigeminal nucleus and the NST in particular. From here, there is potential to activate four core neuromodulatory networks, including the noradrenergic (LC), cholinergic (nucleus basalis of Meynert), serotonergic (RN), and the substantia nigra and ventral tegmental area dopaminergic neurotransmitter systems [57]. These structures, in turn, can profoundly affect the function of different brain networks involved in cognitive functioning, including the thalamus, hypothalamus, hippocampus, amygdala, and cortical areas.

TNS cognitive effects on healthy subjects are much less characterized, both in features and magnitude. However, based on the anatomical and functional data reported above, it can be assumed that the possible effects on the cognitive function of TNS might share the same mediators and CNS targets used by VNS. On this basis, it is possible to hypothesize that paired stimulation of both trigeminal and vagal afferents at the external ear may result in improved results.

Another modality used for activating the trigeminal system is translingual nerve stimulation (TLNS), a neuromodulation approach often used in combination with neurological rehabilitation procedures, to improve outcomes for subjects with neurological conditions possibly by facilitating neuroplasticity-related changes in the brain [98,113,114]. So far, most studies demonstrated the feasibility and safety of TLNS, as well as its usefulness mainly in the treatment of balance and gait deficits in subjects with traumatic brain injury and multiple sclerosis [113,115]. TLNS has also been recently applied, paired with sound stimulation, for tinnitus treatment with ambiguous results [25,116,117]. Interestingly, it has recently been shown that a single 20 min TLNS administration in healthy subjects can affect resting brain activity, as indexed by high-density EEG [118]. This observation, despite substantial differences in the study methods, is also in line with the results of a study performed with fMRI in traumatic brain injury (TBI) patients [119]. These effects on resting brain activity, together with the improvements in cognition demonstrated in TBI patients, suggest that TLNS may be an interesting technique to be evaluated for cognitive effects. With regard to the underlying neurobiological mechanisms, by using TLNS, it is possible to stimulate two cranial nerves: trigeminal (lingual branch) and facial (*chorda tympani* branch). It is hypothesized that the stimulation of these structures modulates the activity of their respective brainstem nuclei, including the NST [115]. In a bottom-up fashion, connected regions are also subsequently affected, modifying corresponding neural network activity.

## 5. Conclusions

The increasing evidence from clinical research of the cognitive effects of TNS has stimulated basic studies aimed at unraveling sites and mechanisms of TNS action at the CNS level. However, these are yet to be understood in both physiological and pathological conditions. Available neurophysiological and behavioral evidence confirms an effect of TNS over CNS activity in healthy subjects. However, with regard to any activity at the cortical level, it cannot be excluded that these effects may be, at least in part, mediated by TNS action on brainstem structures. Since the neurobiological mechanisms by which TNS may act on brainstem structures are not well characterized, the nature of the resulting modulatory effects on supraspinal nuclei and structures remains largely speculative.

One of the most important factors hampering our understanding of TNS effects is the heterogeneity of the methodology of stimulation in both animal and human studies, which makes it difficult to compare results between studies. For instance, rodents have generally been found to be adequate for mechanistic studies of TNS. V1 and V2 divisions (purely sensory branches), in particular, have largely been used as viable access points for either invasive or non-invasive stimulation [120]. One striking disparity in the available literature is the multiple surgical approaches to access the IoN in rodents, which has the potential to become an unnecessary variable [18,61,121,122]. This aspect has been extensively discussed by Dingle and colleagues (2019) [122], where different accesses to the IoN and supraorbital nerve (SoN) are shown, in an effort to standardize the surgical approach as an integral part of the reproducibility of future TNS studies. Initial TNS studies have been performed by implanting cuff electrodes on IoN [18], which is relatively large and easy to access, making it an ideal target either for mechanistic studies or electrode/stimulation procedures development. However, since IoN stimulation is also commonly used to study chronic neuropathic pain in rats [123,124,125], it is essential to ensure that diligent consideration is given to both electrode design and implantation methodology.

In fact, constriction injury to the IoN could result in a trigeminal neuropathic pain syndrome with profound effects on behavior [125]. It has been suggested that trigeminal neuropathic pain may induce profound CNS modification at different levels, inducing reorganization of cortical sensory areas and negatively affecting the function of the dopaminergic mesolimbic system, one of the four core neuromodulatory networks possibly involved in TNS cognitive effects [126,127].

Similarly, in human studies, TNS may be administered in different ways (using subcutaneous, percutaneous, or cutaneous electrodes), for different amounts of time (acute or chronic treatment), and with different stimulation parameters (waveform, amplitude, frequency, etc.). As of today, stimulation parameters are mainly chosen depending on the used device characteristics and the physician’s (or experimenter’s) particular experience. Together with our lack of basic knowledge of the neurobiological effects of TNS, this heterogeneity in administration protocols seriously hampers the comparison of data by different researchers. Another important confounding factor may result from differences in study populations, since the neurobiology of aging-induced cognitive decline may profoundly differ from neurodegeneration-induced cognitive decline, let alone results from healthy subjects. Lastly, the methodology used for cognitive function assessment should also be adequately sensitive to properly define the population under study, as well as specifically designed to allow comparison of the results obtained. To this end, the time-course of the TNS administration protocol must be considered in order to allow a clear distinction between acute effects measured during stimulation, subacute effects measured immediately after, and long-term effects measured after a certain time period. This may be particularly important to differentiate results that could reflect functional changes from structural neuroplastic modifications. With regard to cognitive function assessment, several different approaches can be used, ranging from pen-and-paper tests to more objective neurophysiological methods. In our view, an appropriate combination of these approaches should be used in future studies. In fact, new analysis methods allow combining cognitive test results with data from objective methods, such as functional magnetic resonance, magnetic encephalography, or EEG, in order to monitor different cognitive domains [128,129]. So far, the large heterogeneity of the available data, either in terms of TNS administration protocols and/or populations under study, is both a cause and consequence of our lack of knowledge about the true relevance of TNS effects on cognition. However, new studies aimed to measure specific psychobiological markers after TNS in homogeneous populations should be able to produce relevant results.

Thus, despite being a safe, well-tolerated, and versatile potential intervention, TNS application for cognitive empowering and/or cognitive decline may remain an underrated or overrated approach until, as the field continues to grow, the standardization of practice allows for data comparisons across studies.
